# Non-specific Interactions Between Macromolecular Solutes in Concentrated Solution: Physico-Chemical Manifestations and Biochemical Consequences

**DOI:** 10.3389/fmolb.2019.00010

**Published:** 2019-03-13

**Authors:** Travis Hoppe, Allen P. Minton

**Affiliations:** Laboratory of Biochemistry and Genetics, National Institute of Diabetes and Digestive and Kidney Diseases, National Institutes of Health, Bethesda, MD, United States

**Keywords:** concentrated protein solutions, square well fluid, thermodynamic activity coefficient, light scattering, osmotic pressure, liquid-liquid phase separation

## Abstract

A general thermodynamic formulation of the effect of hard and soft non-specific intermolecular interactions upon reaction equilibria is summarized. A highly simplified quantitative model for non-specific intermolecular interaction is introduced. This model is used to illustrate how the magnitudes of attractive and repulsive components of the overall intermolecular interaction, and the balance between them, influence the concentration-dependent properties of a highly concentrated solution of a single macromolecular solute. The properties calculated using the results of computer simulation and an approximate analytical model are found to agree qualitatively with the results of experimental measurements on protein solutions over a broad range of concentration.

## Introduction

In a complex and highly volume-occupied intracellular or cytomimetic environment, a macromolecule or macromolecular complex within the fluid phase (let us call it the probe) will find itself in the immediate vicinity of other macromolecules of the same or other species. Under such circumstances, interaction between the probe and its macromolecular neighbors is unavoidable. Depending upon the chemical compositions of the probe and the neighbors with which it interacts, the free energy of interaction between the probe and its immediate environment may be net repulsive or net attractive. Variation of the free energy of interaction will have a variety of consequences for the reactivity of the probe and the chemical reactions in which it participates, which we shall review below.

We define interaction between two solute molecules in solution as the existence of a correlation between their positions, orientations, and motions. The molecules may be said to be non-interacting only if the position or motion of the first molecule is entirely unaffected by the presence of the second molecule and vice-versa. At the most basic level this could only be true if the two molecules are separated by a distance that is large relative to molecular dimensions, since clearly the two molecules cannot occupy the same space or pass through each other. Thus, the most basic and universal type of intermolecular interaction is steric, which becomes infinitely repulsive when the surfaces of the two molecules come into contact. In addition, the two molecules may be mutually influenced by each other at longer distances due to the presence of electrostatic or solvent-mediated interactions (Minton, 1983, 2013). Longer-ranged interactions may be strong and highly dependent upon the mutual orientations of the two molecules, in which case they are referred to as specific, and typically result in the formation of experimentally characterizable static or dynamic complexes with a defined structure and a lifetime that depends upon the free energy of association and the kinetics of dissociation. However, under physiological conditions, longer-ranged interactions between two functionally unrelated molecules are likely to be weak and independent, or only weakly dependent, upon mutual orientation of the two molecules, and do not lead to the formation of specific complexes. In the present work, we shall concern ourselves only with non-specific interactions, namely the steric, or “hard” interaction, and longer-ranged weak or “soft” interactions. Modulation of equilibria governing specific biochemical reactions by side reactions resulting from of specific interactions between reactant and environmental solutes is treated elsewhere (Rivas and Minton, 2017).

In the following section we shall summarize a general thermodynamic formulation of the effect of hard and soft non-specific intermolecular interactions upon reaction equilibria. We then introduce the square well-potential and extended Kihara model for non-specific intermolecular interactions. Then computer simulations of a square well-fluid and the analytical extended Kihara model will be used to calculate the concentration dependence of several experimentally measurable properties of a macromolecular solution, and the results of the two sets of calculations compared. Finally, the calculated properties are compared with the results of measurements in the literature.

## The Thermodynamic Activity Coefficient and Chemical Equilibria

Chemical equilibrium constants are commonly written as a function of the concentrations of reactants and products. Contrary to common perception, these quantities are not true constants at constant temperature and pressure. Let us consider two simple examples; a general treatment is provided elsewhere (Zimmerman and Minton, 1993).

Example 1. The simple reversible transition between the native (*N*) and fully unfolded (*U*) conformations of a globular protein

(1) N⇆U

This reaction is characterized by the equilibrium unfolding constant *K*_*NU*_. It may be shown (Zimmerman and Minton, 1993) that at constant temperature and pressure

(2a) $$K_{NU}\equiv \frac{c_{U}}{c_{N}}=K_{NU}^{o}\Gamma_{NU}$$

where,

(2b) $$\Gamma_{NU}=\gamma_{N}/\gamma_{U}\;$$

Here $K_{NU}^{o}$ denotes the true thermodynamic equilibrium constant, dependent only upon temperature and pressure, Γ_*NU*_ a non-ideality or “crowding” factor, *c*_*i*_ and γ_*i*_ the molar concentration and thermodynamic activity coefficient of species *i*, respectively. Activity coefficients will be shown below to be functions of solute-solute interaction and hence dependent in principle upon the concentrations of all solute species in the solution.

Example 2. A simple reversible bimolecular association or binding reaction

(3) A+B⇆AB

This reaction is characterized by the equilibrium association constant *K*_*AB*_. As in the case of Example 1, it may be readily shown (Zimmerman and Minton, 1993) that at constant temperature and pressure

(4a) $$K_{AB}\equiv \frac{c_{AB}}{c_{A}c_{B}}=K_{AB}^{o}\Gamma_{AB}$$

where,

(4b) $$\Gamma_{AB}=\gamma_{A}\gamma_{B}/\gamma_{AB}$$

Here $K_{AB}^{o}$ denotes the true thermodynamic equilibrium constant, dependent only upon temperature and pressure, Γ_*AB*_ the non-ideality or “crowding” factor and γ_*A*_, γ_*B*_, and γ_*AB*_ the thermodynamic activity coefficients of the respective species.

The thermodynamic activity coefficient of solute species *i* is a measure of the free energy of interaction of a molecule of that species and all of the other solute molecules in solution at equilibrium. According to the solution theory of McMillan and Mayer (McMillan and Mayer, 1945), the thermodynamic activity coefficient of an individual macromolecular solute species may be expressed as a power series in the concentrations of all macromolecular solute species as follows:

(5) $$\ln\gamma_{i}=\sum_{j}B_{ij}c_{j}+\sum_{j}\sum_{k}B_{ijk}c_{j}c_{k}+...$$

where *B*_*ij*_ and *B*_*ijk*_, respectively, denote two-body and three-body interaction coefficients that are independent of macrosolute solute composition at fixed temperature and pressure. These interaction coefficients are defined functions of the potential of mean force^1^ acting between molecules of species *i* and *j*. For a solution containing a single macromolecular solute species, Equation (5) reduces to

(6) $$\ln\gamma =B_{2}c+B_{3}c^{2}+...$$

It follows from Equations (5) and (6) that as the solution becomes progressively more dilute and all *c*_*i*_ → 0, all γ_*i*_ → 1, so that $K_{NU}\to K_{NU}^{0}$ and $K_{AB}\to K_{AB}^{0}$. As solute concentrations increase, and solute molecules are on average closer together, the activity coefficients of one or more solute species may diverge substantially from unity, and the crowding factors Γ_*NU*_ and Γ_*AB*_ may deviate from unity by as much as several orders of magnitude. Experimental confirmation of this expectation is widespread and has been tabulated in several reviews (Minton, 1983; Zimmerman and Minton, 1993; Hall and Minton, 2003; Zhou et al., 2008).

It follows from Equations (2) and (4) that conformational and association equilibria may depend significantly upon the concentrations of environmental macromolecules as well as the concentrations of reactant(s) and product(s).

## Experimentally Observable Manifestations of Non-specific Intermolecular Interactions

The concentration dependence of several experimentally measurable solution properties are directly related to the concentration dependence of the activity coefficient of solute. The properties are:

(1) The average intensity of light scattered from a protein solution:

(7) $$I\left(c\right)=\alpha \frac{c}{1+c\left(d\ln\gamma /dc\;\right)}$$

(2) The apparent molar mass of a solute determined from its radial concentration gradient in a centrifuge cell spinning at constant rotor speed and temperature at sedimentation-diffusion equilibrium:

(8) $$M_{app}\left(c\right)\equiv \beta \frac{d\ln c}{dx^{2}}=\frac{M}{1+c\left(d\ln\gamma /dc\;\right)}$$

where *x* denotes distance from the center of rotation.

(3) The osmotic pressure of a solution:

(9) $$\Pi \left(c\right)=\lambda \left[c+\int_{0}^{c}c^{*}\left(\frac{d\ln\gamma }{dc^{*}}\right)dc^{*}\right]$$

where α, β, and λ denote method-specific constants of proportionality (Tanford, 1961; Cantor and Schimmel, 1980). Given experimental data of sufficient accuracy and precision describing the concentration dependence of any of these properties, one may in principle invert the appropriate equation given above to obtain the concentration-dependence of ln γ (Fodeke and Minton, 2010; Wu and Minton, 2015). In the following sections, we present a simplified theoretical model for the potential of mean force, and utilize this model to calculate the concentration dependence of ln γ and several experimentally observable properties. Then results of calculations of these concentration-dependent properties are compared with results of experimental measurements carried out on protein solutions.

## Square Well Potential–A Simple Description of Non-specific Interactions

If a highly simplified model of intermolecular interaction properly captures essentials of the actual intermolecular interaction, one would expect it to qualitatively reproduce observed behavior and systematic trends. It follows that if the model does successfully reproduce observed behavior, one may have some confidence that the model assumptions are at least qualitatively correct.

Our investigation therefore starts with a simple model for the potential of mean force acting between globular macromolecules in solution. The first protein solutions to be quantitatively characterized at high concentration were solutions of hemoglobin (Adair, 1928; Williams, 1973; Ross et al., 1978). Analysis of the concentration dependence of osmotic pressure and sedimentation equilibrium of hemoglobin solutions led to the conclusion that solute-solute interactions between hemoglobin molecules in solutions of moderate ionic strength were exclusively repulsive, and that solution properties could be accounted for by a model in which the protein molecule was represented by a hard spherical particle of approximately the same size and shape as the hemoglobin molecule (Ross and Minton, 1977). Subsequent experimental studies of the high concentration behavior of solutions of other proteins revealed that hemoglobin was a rather special case of a purely steric interaction, and that more generally, protein molecules interacted with each other not only via steric repulsion, but also via electrostatic and other longer-ranged interactions that could be either primarily attractive or repulsive, with a magnitude that depends upon experimental conditions (Minton and Edelhoch, 1982; Minton, 1995; Jiao et al., 2010; Sarkar et al., 2014; Guseman et al., 2018). Hence any general model for protein-protein interaction must allow for contributions from both steric repulsion and longer-ranged repulsion or attraction. The simplest model taking both features into account is the square well (SW) potential of mean force, defined as follows.

**Figure 1:**
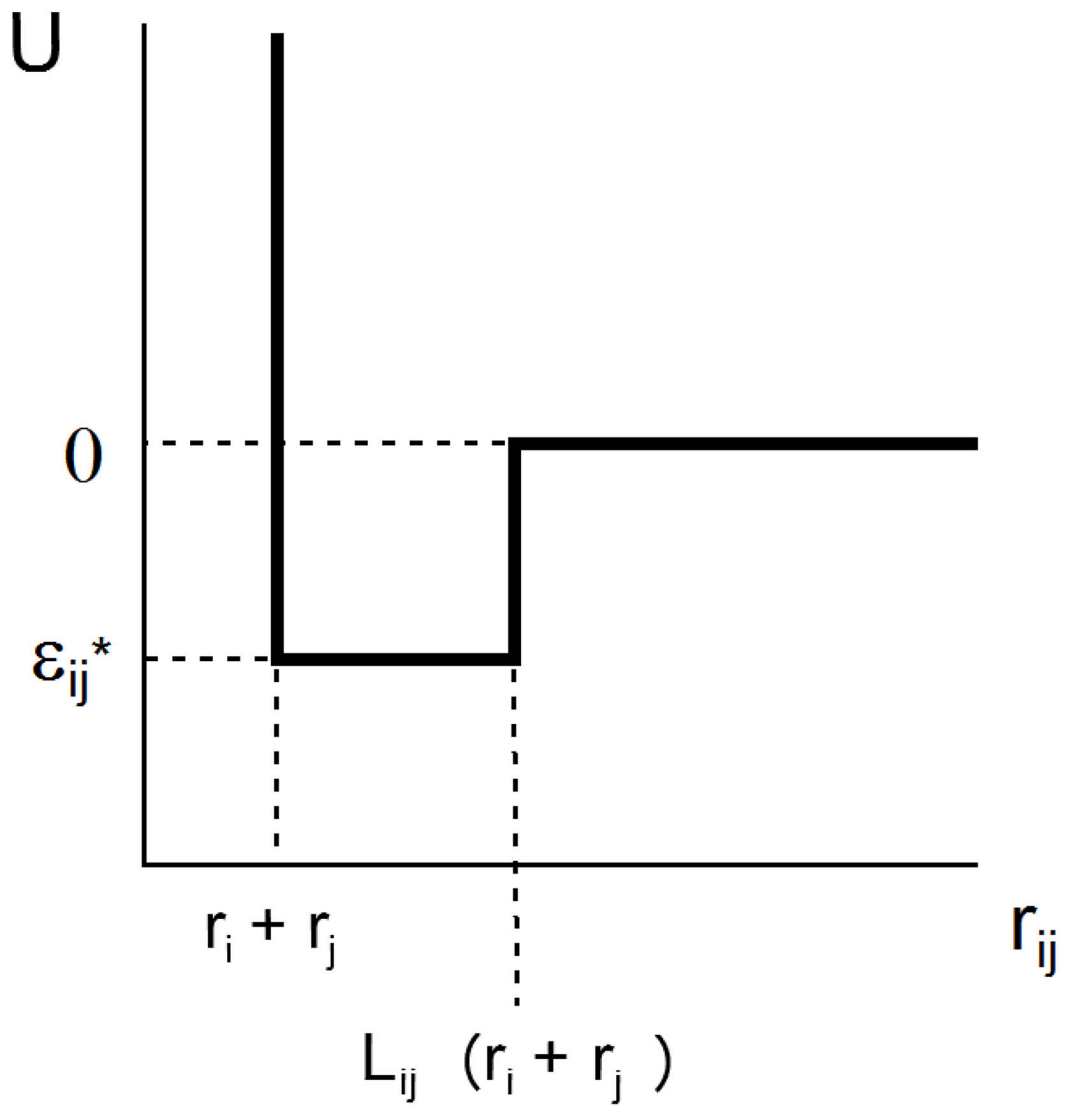
Plot of square well-potential of mean force as a function of center-to-center distance between two interacting spherical solute molecules.

The SW potential of mean force acting between two spherical solutes of radii *r*_*i*_ and *r*_*j*_ separated by intercenter distance *r*_*ij*_ is characterized by two parameters, *L* and ε, that define the range and depth of the attractive square well.

(10) $$U(r_{ij})=\{\begin{array}{l} \infty \;r_{ij}<r_{i}+r_{j}\\ \varepsilon /kT\;r_{i}+r_{j}\leq r_{ij}<L_{ij}\left(r_{i}+r_{j}\right)\\ 0\;L_{ij}\left(r_{i}+r_{j}\right)\leq r_{ij}\; \end{array}$$

where *k* denotes Boltzmann's constant and *T* the absolute temperature. This potential is schematically depicted in Figure 1. For ease of notation, we shall subsequently denote ε/*kT* by ε^*^, indicating that this value of ε is expressed in units of the thermal energy *kT*. In the case of a solution containing only a single macromolecular solute, *r*_*i*_ = *r*_*j*_ = *r* and *L*_*ij*_ = *L*.

## Estimation of the Composition Dependence of ln γ in a Square Well Fluid

Our ultimate goal is to develop a qualitatively realistic analytical model for estimation of the concentration dependence of the thermodynamic activity of each solute species in a fluid of particles interacting via square well-potentials. Here we compare the results of calculations performed using an approximate analytical model developed previously (Hoppe and Minton, 2016) with numerical results obtained via computer simulation. We shall subsequently refer to this publication as HM.

Using the method of discrete molecular dynamics as described in HM, simulations of equilibrium square well fluids were performed at fractional volume occupancies of up to 0.32 for various values of the range parameter *L* > 1.25 and the depth parameter 0 ≤ ε* ≤ −1.5. The value of ln γ was then calculated via the method of Widom insertion, as described in HM. The dependence obtained from simulations are regarded as standards to which we shall compare the approximate estimates described below.

In the relations described below, the concentration dependence of activity coefficients and colligative properties of solutions may be expressed as functions of the molar concentration of solute *c* or the unitless fraction of solution volume occupied by solute ϕ. The choice of unit is a matter of convenience in numerical computation. We note that these quantities are proportional to each other and may be readily interconverted^2^ according to

(11) $$\phi =cM\bar{v}/1000\;$$

where *M* denotes the molar mass and $\bar{v}$ the specific exclusion volume in cm^3^/g.

Kihara derived exact analytical relations for the second and third osmotic virial coefficients of a multicomponent square well fluid (Kihara, 1953, 1955). The second and third osmotic coefficients are, respectively, proportional to the two- and three-body interaction coefficients in Equation (5) (Hoppe and Minton, 2016). In HM it was observed that at fractional volume occupancies exceeding ϕ = 0.15, values of ln γ calculated using the Kihara model became progressively more negative (or less positive) than those obtained from the computer simulations, indicating the limits of a calculation that takes into account explicitly only two- and three-body interactions. In order to compensate for the underestimate of ln γ at higher concentrations, we therefore proposed an approximate extension, previously referred to as the hybrid model, but which we shall henceforth refer to as the extended Kihara or Kihara+ model. According to this approximate treatment, the thermodynamic activity coefficient is partitioned into contributions from hard core steric repulsion and longer-ranged non-specific “soft” interactions:

(12) $$\ln\gamma \left(c\right)=\ln\gamma_{hard}\left(c\right)+\ln\gamma_{soft}\left(c\right)$$

Theories of hard sphere fluids provide quantitative treatments of steric repulsive interactions of hard spherical particles that have been shown to be quite accurate at fractional volume occupancies up to 0.5 (Minton, 1998). We accordingly calculate the contribution from steric repulsion utilizing results obtained from the scaled particle theory of hard sphere fluids (Lebowitz et al., 1965):

(13) $$\ln\gamma_{hard}\left(c\right)=\ln\gamma_{SPT}\left(c\right)$$

**Figure 2:**
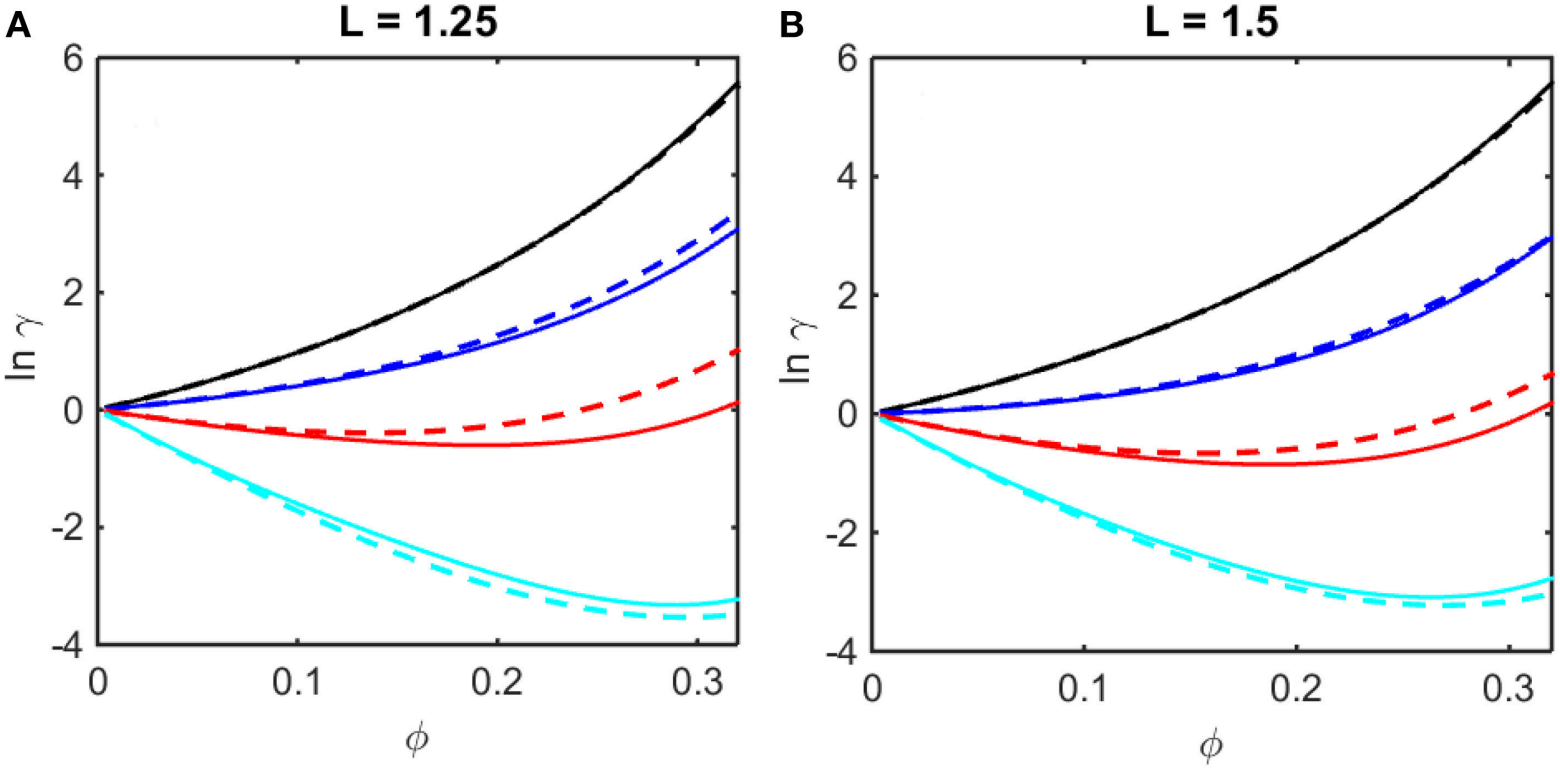
Concentration dependence of ln γ in a square well fluid obtained from computer simulation (solid curves) and calculated using the Kihara+ model (dashed curves). **(A)** L = 1.25, with ε^*^ = 0 (black), −0.5 (blue), −1.0 (red), and −1.5 (cyan). **(B)** L = 1.5, with ε^*^ = 0 (black), −0.3 (blue), −0.6 (red), and −0.9 (cyan).

The contribution from non-specific attraction is then calculated according to

(14) $$\ln\gamma_{soft}≅B_{2,soft}c+B_{3,soft}c^{2}$$

where coefficients *B*_2,*soft*_ and *B*_3,*soft*_ in Equation (14) are obtained from the Kihara expressions for *B*_2_ and *B*_3_, respectively, by eliminating the contributions from steric repulsion in each expression (Hoppe and Minton, 2016). The concentration dependence of ln γ upon ϕ calculated using Equations (12–14) are plotted in Figures 2A,B together with results of computer simulation. It is evident that the Kihara+ model provides a better than qualitative estimate of the concentration dependence of ln γ over the entire range of ϕ encompassed by computer simulations, and for values of ε* spanning the range between fully repulsive to partly repulsive to predominantly attractive interparticle interactions.

It is observed that a similar dependence of the activity coefficient upon volume fraction is obtained for combinations of a larger value of *L* and a less negative value of ε^*^ (for example, compare the dependences calculated for *L* = 1.25 and ε* = −1.5 in panel A and those calculated for *L* = 1.5 and ε^*^ = −0.9 in panel B). This is to be expected, as it indicates that concentration-dependent activity is dependent upon integrals over the entire potential function, e.g., *B*_2_ and *B*_3_ (McMillan and Mayer, 1945), rather than upon the value of an individual parameter in the potential function. In the following sections we shall compare calculations of the colligative properties of solutions calculated utilizing the results of simulations and the Kihara+ model.

## Colligative Properties of a Square Well Fluid

Inspection of Equations (2–4) reveals that all three colligative properties depend upon the concentration dependence of *c*(*d* ln γ/*dc*) = ϕ(*d* ln γ/*dϕ*). Calculation of this quantity is facilitated by the observation that ln γ may be well-described over the range 0 < ϕ ≤ 0.4 by the empirical polynomial

(15) $$\ln\gamma =Q_{1}\phi +Q_{2}\phi^{2}+Q_{3}\phi^{3}+Q_{4}\phi^{4}$$

where the coefficients are obtained by linear least-squares modeling of the results of simulation or model calculations, as shown in Supplementary Information, Appendix 1. It follows that

(16) $$\begin{array}{l} c\left(d\ln\gamma /dc\;\right)=\phi \left(d\ln\gamma /d\phi \;\right)=Q_{1}\phi \;\\ \;+2Q_{2}\phi^{2}+3Q_{3}\phi^{3}+4Q_{4}\phi^{4} \end{array}$$

and

(17) $$\begin{array}{l} \int_{0}^{c}c^{*}\left(\frac{d\ln\gamma }{dc^{*}}\right)dc^{*}=\int_{0}^{\phi }\phi^{*}\left(\frac{d\ln\gamma }{d\phi^{*}}\right)d\phi^{*}=\frac{1}{2}Q_{1}\phi^{2}\;\\ \;+\frac{2}{3}Q_{2}\phi^{3}+\frac{3}{4}Q_{3}\phi^{4}+\frac{4}{5}Q_{4}\phi^{5} \end{array}$$

**Figure 3:**
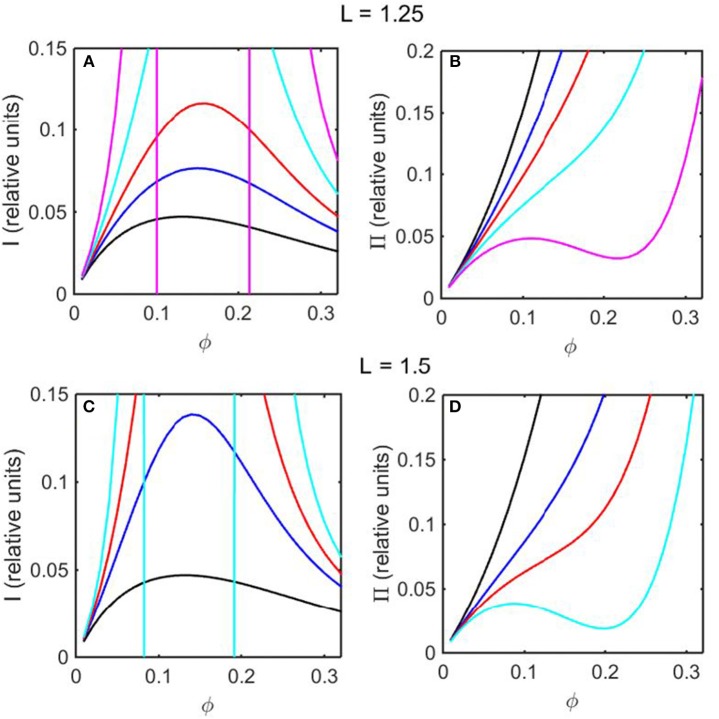
Colligative properties of square well-solutions as calculated from computer simulation. Concentration dependence of scattered light intensity *I* for L = 1.25 **(A)** and L = 1.5 **(C)**. Concentration dependence of osmotic pressure Π for L = 1.25 **(B)** and L = 1.5 **(D)**. Values of ε^*^ used for L = 1.25 calculations were 0 (black), −0.55 (blue), −0.8 (red), −1.05 (cyan), and −1.3 (magenta). Values of ε^*^ used for L = 1.5 calculations were 0 (black), −0.5 (blue), −0.65 (red), and −0.8 (cyan).

**Figure 4:**
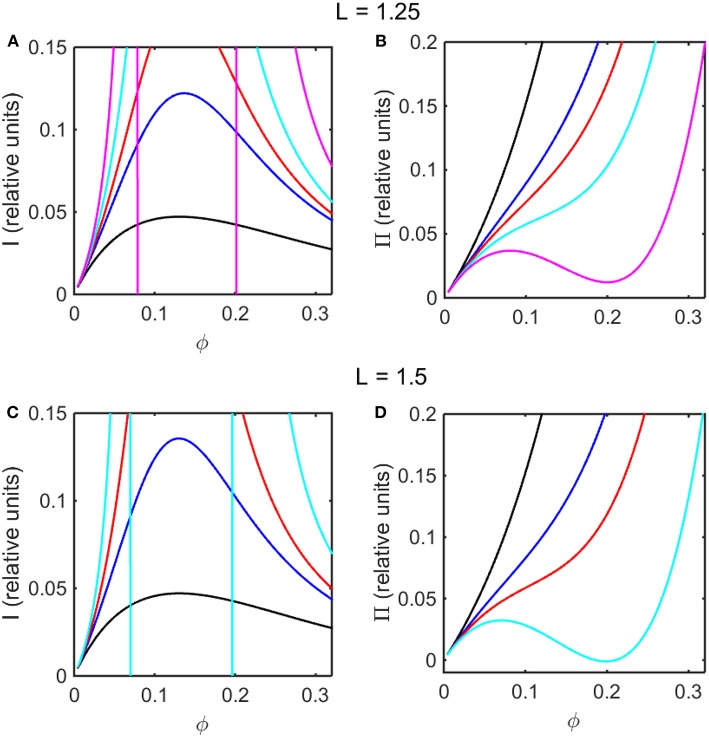
Colligative properties of square well-solutions as calculated from Kihara+ model. Concentration dependence of scattered light intensity *I* for L = 1.25 **(A)** and L = 1.5 **(C)**. Concentration dependence of osmotic pressure Π for L = 1.25 **(B)** and L = 1.5 **(D)**. Values of ε^*^used for L = 1.25 calculations were 0 (black), −0.95 (blue), −1.1 (red), −1.25 (cyan), and −1.4 (magenta). Values of ε^*^used for L = 1.5 calculations were 0 (black), −0.55 (blue), −0.7 (red), and −0.85 (cyan).

Using the concentration dependence of ϕ(*d* ln γ/*dϕ*) obtained by modeling results of the computer simulations together with Equations (15–17), the dependence of scattered light intensity and the osmotic pressure upon ϕ, calculated using Equations (2) and (4) with *L* = 1.25 and selected values of ε ^*^ are plotted in Figures 3A,B, and calculated with *L* = 1.5 and selected values of ε^*^ are plotted in Figures 3C,D. Using the concentration dependence of ϕ(*d* ln γ/*dϕ*) = *c*(*d* ln γ/*dc*) obtained from the Kihara+ model and modeled using Equations (15–17), the dependence of scattered light intensity and the osmotic pressure uponϕ, calculated using Equations (2) and (4) with *L* = 1.25 and selected values of ε^*^ are plotted in Figures 4A,B, and calculated with *L* = 1.5 and selected values of ε^*^are plotted in Figures 4C,D.

In the absence of intersolute interactions (i.e., in the limit of high dilution), the intensity of static light scattering is linear in concentration (Cantor and Schimmel, 1980). In Figures 3A,C, 4A,C, it is observed that in the presence of repulsive steric interaction only (ε^*^ = 0; black curves), the initial slope of the concentration-dependent scattered light intensity decreases monotonically with increasing concentration. In the same figures it is observed that when longer-ranged attraction is added to the steric repulsion (ε^*^ < 0; colored curves) the initial slope of the concentration-dependent scattered light intensity increases with increasing depth of the square well. For well-depths less negative than a certain critical value, which we will refer to as $\varepsilon_{crit}^{*}$, the scattering reaches a maximum with increasing ϕ and subsequently decreases with further increases in ϕ. Such behavior is observed in concentrated protein solutions (Fernández and Minton, 2008; Scherer et al., 2010; Scherer, 2015). When the value of ε^*^ becomes more negative than $\varepsilon_{crit}^{*}$, indicating stronger solute-solute attraction, the slope of the curve of I vs. ϕ increases monotonically, and diverges (I → ∞) at a value of ϕ such that *c*(*d* ln γ/*dc*) = ϕ(*d* ln γ/*dϕ*) = −1, indicated by a vertical line in the plot. Divergence of scattering is observed experimentally by the rapid onset of turbidity or opalescence (Taratuta et al., 1990; Raut and Kalonia, 2015), and indicates the existence of a liquid-liquid phase transition. In Figures 3B,D, 4B,D it is observed that when ε^*^ becomes more negative than $\varepsilon_{crit}^{*}$ the concentration dependence of the calculated osmotic pressure exhibits non-monotonic behavior, which is physically unrealizable, and is likewise indicative of a phase transition, which may be characterized by analyzing the non-monotonic behavior as described in Supplementary Information, Appendix 2 and the following section.

Upon comparison of the results shown in Figures 3, 4 it is evident that the concentration-dependent colligative properties calculated from the Kihara+ model are very similar to those calculated from the computer simulations, and differ only quantitatively. The significance of this resemblance will be discussed in the concluding section of this report.

## Liquid-Liquid Phase Separation in a Square Well Fluid

Proteins may exist in two (or more) phases when the chemical potential of the protein is equal in both phases. We are all familiar with the equilibrium between the solution and solid (crystalline) phases manifested as finite solubility. Biochemists are less familiar with the equilibrium between two immiscible solution phases containing the same protein at two different concentrations, known as liquid-liquid phase separation or LLPS. This phenomenon has been observed experimentally in solutions of several proteins under specific conditions (Taratuta et al., 1990; Mason et al., 2010; Reiche et al., 2017), and is thought to be responsible for the formation of liquid-like globules enriched in a specific protein within cellular cytoplasm (Shin and Brangwynne, 2017).

**Figure 5:**
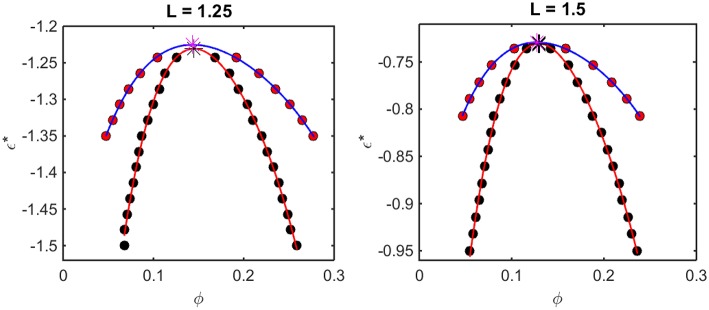
Phase diagrams calculated from computer simulation. Red symbols: points along the binodal or coexistence curve. Blue curve: best polynomial fit to binodal points. Black symbols: points along the spinodal curve. Red curve: best polynomial fit to spinodal points. For L = 1.25, ϕ_*crit*_ = 0.16 and $\varepsilon_{crit}^{*}$ = −1.23, and for L = 1.5, ϕ_*crit*_ = 0.13 and $\varepsilon_{crit}^{*}$ = −0.74.

**Figure 6:**
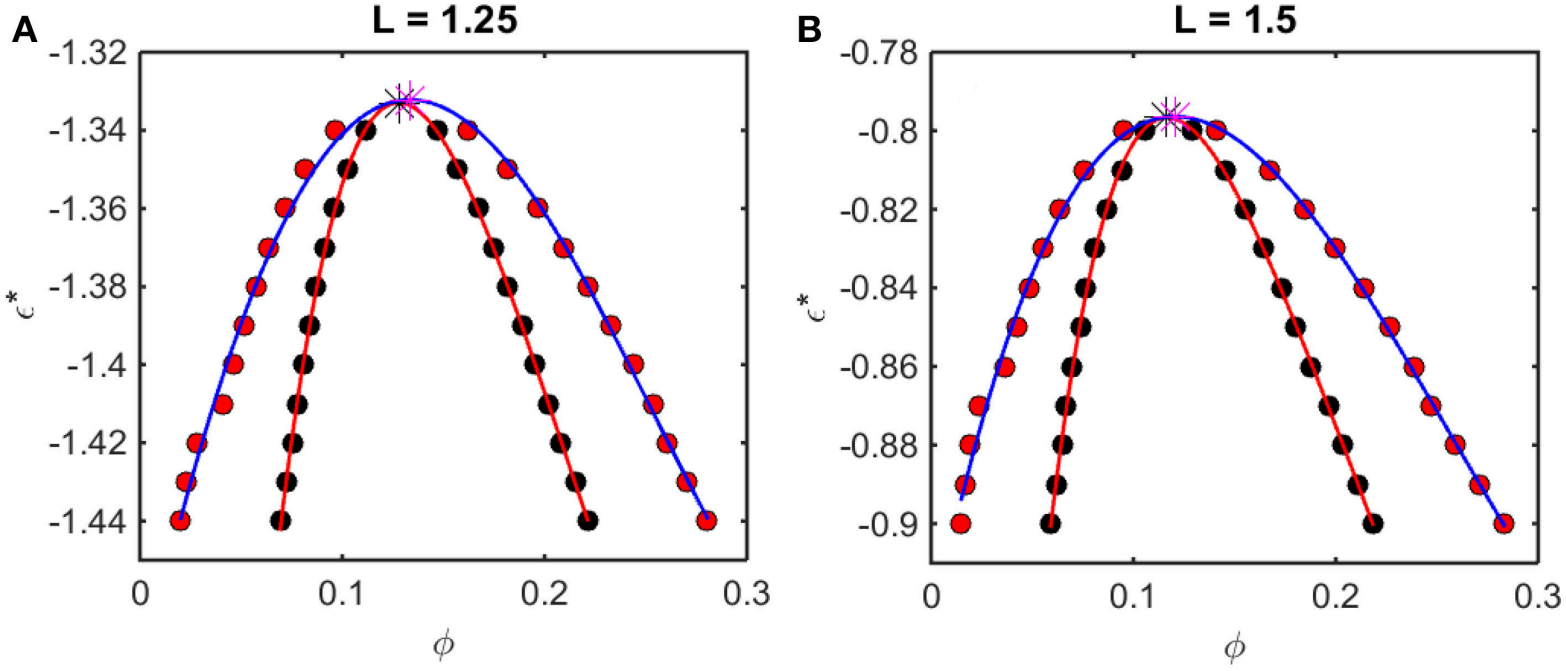
Phase diagrams calculated from Kihara+ model. Symbols and curves as in Figure 5. For **(A)** L = 1.25, ϕ_*crit*_ = 0.13, and $\varepsilon_{crit}^{*}$ = −1.33, and for **(B)** L = 1.5, ϕ_*crit*_ = 0.12 and $\varepsilon_{crit}^{*}$ = −0.80.

According to the McMillan-Mayer theory of solutions (McMillan and Mayer, 1945), the solubility equilibrium is thermodynamically analogous to the equilibrium between a gas and a solid phase, and LLPS is thermodynamically analogous to the equilibrium between the gas and a liquid phase. Thus, the relationship between intermolecular interaction and LLPS may be quantified using formalism developed for analyzing the predicted effect of attractive interactions upon the gas-liquid equilibrium. For given values of *L* and ε^*^, one may calculate the concentration-dependent osmotic pressure as described above. If the concentration-dependent osmotic pressure exhibits non-monotonic behavior, the analysis described in Supplementary Information, Appendix 2 will yield the upper and lower compositions of the two phases at equilibrium, and the upper and lower concentrations corresponding to the limits of metastability of a one-phase solution. When this analysis is performed for a single value of *L* and multiple values of ε^*^, a phase diagram may be obtained by plotting these concentrations as a function of ε^*^. Phase diagrams so constructed using results obtained from computer simulation are plotted in Figure 5, and phase diagrams constructed in the same manner using results obtained using the Kihara + analytical model are plotted in Figure 6.

The outer curve (red symbols and the best-fit polynomial drawn through them) represents the equilibrium coexistence curve, or binodal, and the inner curve (black symbols and best-fit polynomial) represents composition limits of metastability of a single phase solution, or spinodal. For comparison with experimentally measured phase diagrams (see for example Reiche et al., 2017), it should be noted that a decrease in the absolute value of ε^*^ (i.e., an increase in the value of the ordinate) corresponds to an increase in the temperature^3^ or the concentration of any cosolute, such as salt^4^, that weakens the non-specific attractive intermolecular interaction between protein molecules.

For any given value of ε^*^, the value of ϕ lying on the ascending side of the binodal represents the equilibrium concentration of solute in the dilute phase, $\phi_{dil}^{eq}$, and the value of ϕ lying on the descending side of the binodal represents the equilibrium concentration of solute in the concentrated phase, $\phi_{conc}^{eq}$. The value of ϕ lying on the ascending side of the spinodal represents the maximum concentration of a single-phase dilute solution that may exist metastably, even though it is not at equilibrium, $\phi_{lower}^{*}$, and the value of ϕ lying on the descending side of the spinodal represents the minimum concentration of a single-phase concentrated solution that may exist metastably, $\phi_{upper}^{*}$. The apices of the binodal and spinodal curves (or rather the best polynomial fits through the calculated points) converge at a composition and characteristic value of ε^*^ referred to as the critical point. At values of ε^*^ more positive than that at the critical point (attained at higher temperature ^3^ or salt concentration^4^), the solution will exist as a single phase at all concentrations. At values of ε^*^ more negative than that at the critical point (lower temperature or salt concentration), solutions with $\phi \leq \phi_{dil}^{eq}$ or $\phi \geq \phi_{conc}^{eq}$ will exist as a single phase of uniform concentration. Solutions with a total concentration $\phi_{dil}^{eq}<\phi <\phi_{conc}^{eq}$ will exist *at equilibrium* as a mixture of dilute and concentrated phases of fixed composition, where the volume fraction of the concentrated phase will be given by

(18) $$f_{conc}=\frac{\phi -\phi_{dil}^{eq}}{\phi_{conc}^{eq}-\phi_{dil}^{eq}}$$

If the total concentration lies between $\phi_{dil}^{eq}$ and $\phi_{lower}^{*}$, or between $\phi_{upper}^{*}$ and $\phi_{conc}^{eq}$, the solution may exist as a single metastable phase, but depending upon the kinetics of the transition, will eventually demix to form the two phases coexisting at equilibrium^5^.

We observe that the binodal curves calculated from the computer simulations are significantly broader than those calculated from the Kihara+ model, although the spinodal curves are similarly shaped. The reason for this is that the osmotic pressure calculated using the Kihara+ model at very high concentration is systematically greater than that calculated from the simulations. Thus, the high end of the coexistence curve calculated using this model is artifactually shifted toward lower concentrations. Since the divergence between the simulation and model calculations is only significant at the highest concentrations, it may be seen that the spinodal curves and the values of ϕ_*crit*_ and $\varepsilon_{crit}^{*}$ calculated from the Kihara+ model are very close to those calculated from the computer simulations.

## Comparison of Results of Model Calculations to Experimental Measurement

The utility of a simplified model potential of mean force may be judged by its ability to qualitatively account for or predict the observed behavior of a protein solution. Below we present comparisons of the results of model calculations with experimental observations.

### Light Scattering

**Figure 7:**
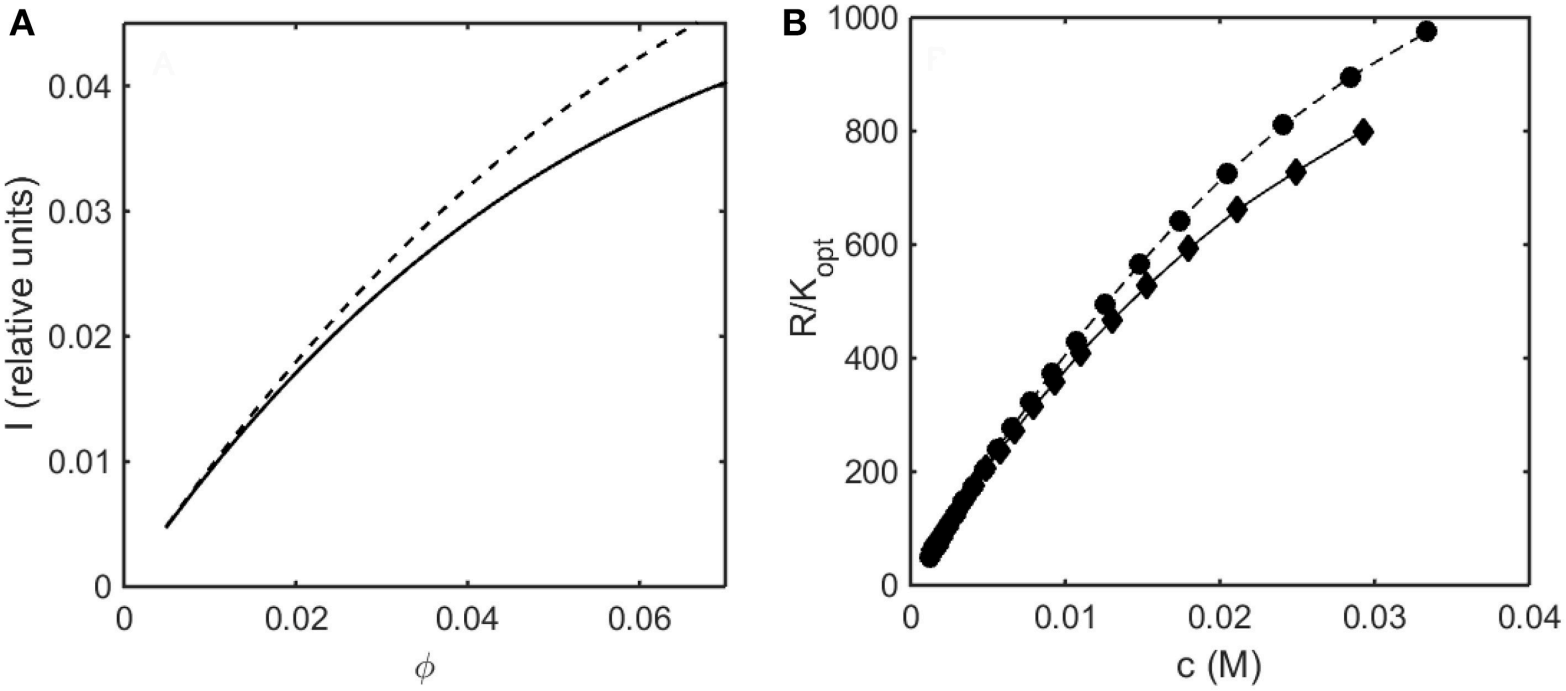
**(A)** Concentration dependence of scattering intensity calculated using the Kihara+ model with L = 1.25 and ε^*^ = 0 (solid curve), and ε^*^ = −0.3 (dashed curve). **(B)** Experimentally measured dependence of scattering intensity of ovalbumin in 10 mM phosphate buffer, pH 7, in the absence (diamonds) and the presence of 0.15 M NaCl (circles). Data of Wu and Minton (2015).

In Figure 7A the relative intensity of light scattering calculated using the Kihara+ model is plotted as a function of concentration for two values of ε^*^ representing no attractive and somewhat attractive attractive intermolecular interaction. In Figure 7B we plot the measured intensity of light scattering as a function of concentration for ovalbumin at pH 7 in low and moderate ionic strength solutions. The increase in ionic strength results in a decrease in the strength of repulsive electrostatic intermolecular interactions, which has the same effect as an increase in the strength of attractive interactions, namely an increase in the scattering intensity.

### Osmotic Pressure

**Figure 8:**
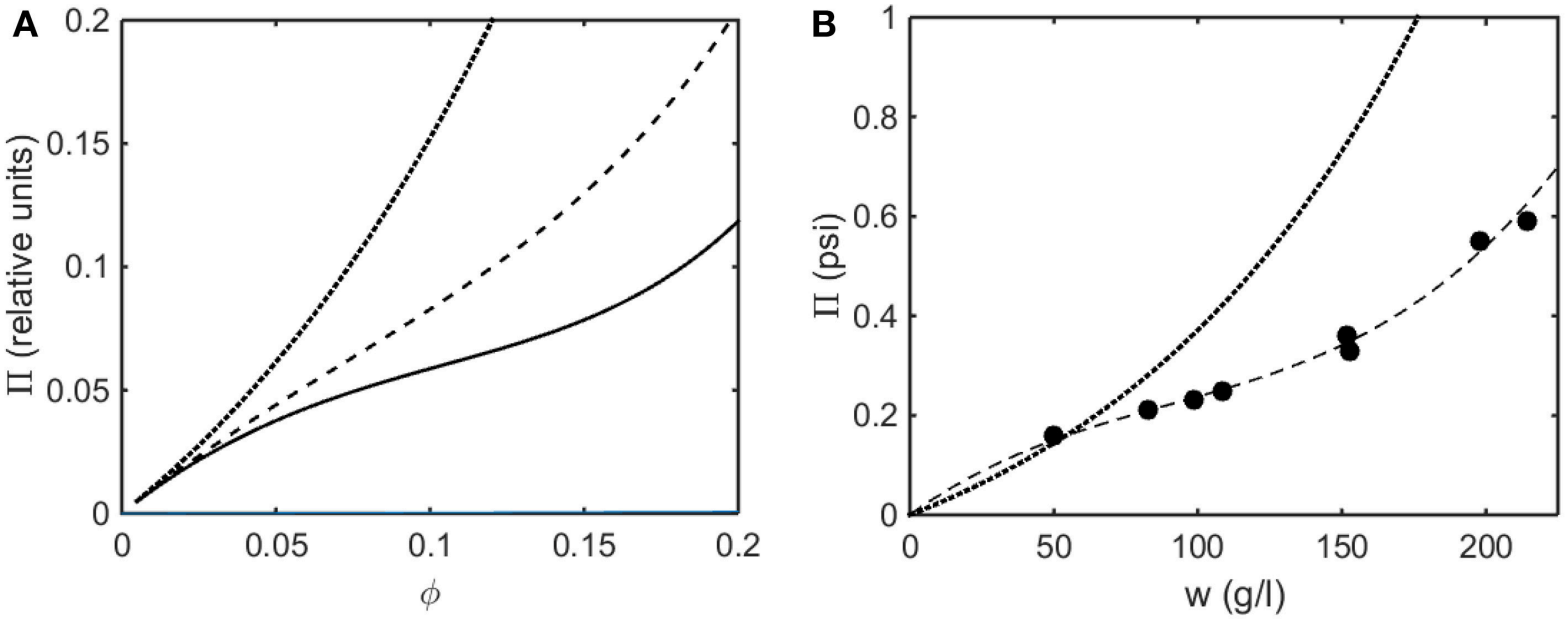
**(A)** Osmotic pressure calculated using Kihara+ model with L = 1.25 and ε^*^ = 0 (dotted), −1.2 (dashed), and −1.4 (solid). **(B)** Experimentally measured osmotic pressure of IgG at pH 7.0. Symbols, data of Yousef et al. (1988). Dashed line is the linear least squares best fit of a cubic polynomial with (0,0) intercept, to guide the eye. Dotted line is calculated assuming molar mass of 65,500 and ε^*^ = 0 (i.e., no attractive interactions).

In Figure 8A the osmotic pressure calculated using the Kihara+ model is plotted as a function of concentration for three values of ε^*^representing different strengths of attractive interaction. In Figure 8B the experimentally measured osmotic pressure of immunoglobulin G is plotted as a function of concentration, together with the dependence of the osmotic pressure upon concentration calculated for the same molar mass in the absence of an attractive interaction (i.e., pure hard steric repulsion). The plot of experimental data displays an inflection point similar to that calculated using the Kihara+ model and a value of ε^*^ slightly less in magnitude than the value at which a phase separation appears.

### Liquid-Liquid Phase Separation

**Figure 9:**
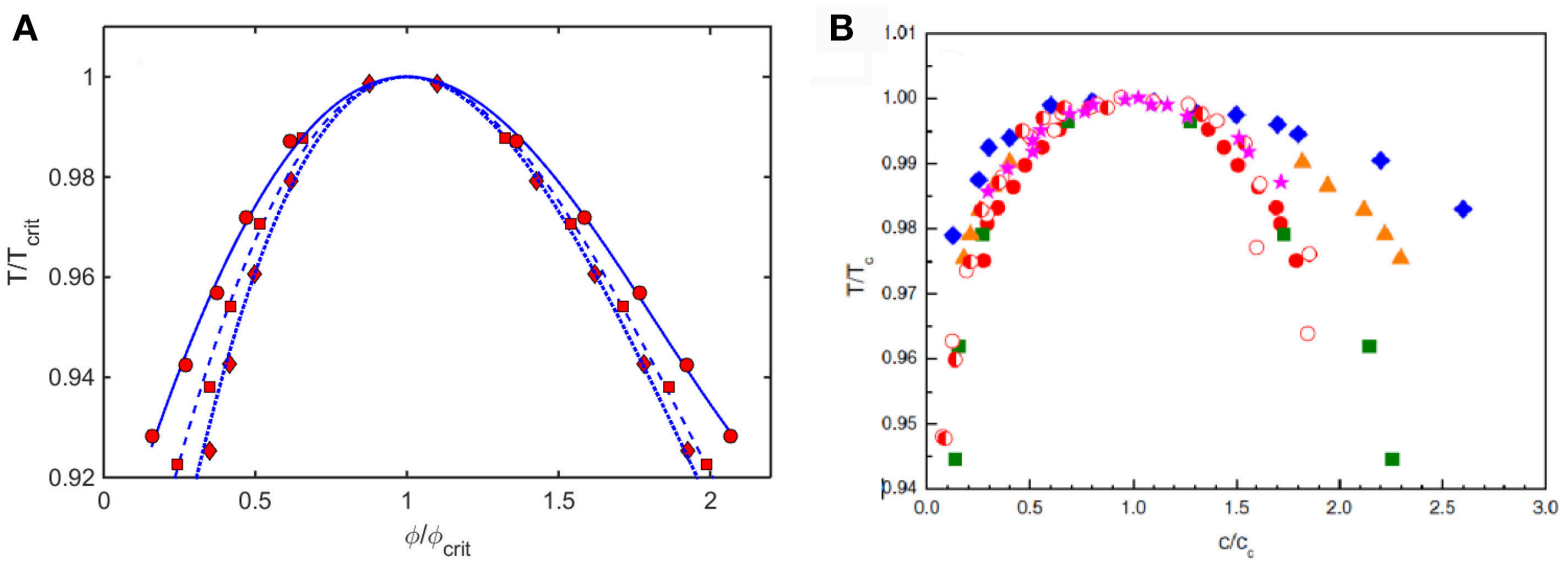
**(A)** Plot of coexistence curves (binodals) calculated using Kihara+ model for L = 1.25 (diamonds, dotted curve), 1.5 (squares, dashed curve), and 1.75 (circles, solid curve). Results are scaled to ϕ_*crit*_ and *T*_*crit*_. **(B)** Plot of scaled coexistence curves of crystallins, lysozyme, and monoclonal antibodies measured under various experimental conditions (Reiche et al., 2017). Figure reprinted from Reiche et al. (2017) with permission by Elsevier.

Coexistence (binodal) curves calculated using the Kihara+ model plotted as ε^*^ against coexistence compositions, such as those shown in Figure 6, may be converted to coexistence curves plotted as a relative temperature against coexistence compositions, where $T_{rel}\equiv -1/\varepsilon^{*}$. Coexistence curves calculated in this manner for three values of *L* and scaled relative to ϕ_*crit*_ and *T*_*crit*_ are plotted in Figure 9A. These are compared to similarly scaled coexistence curves obtained via experimental measurement on various proteins under different experimental conditions plotted in Figure 9B.

## Discussion

One of the objectives of the present work is to demonstrate that computationally demanding atomistically detailed Monte Carlo or Brownian Dynamics simulations are not required to obtain a basic understanding of the major contributions to non-specific interactions between protein molecules in solution. The square well-potential is the simplest model for a potential of mean force containing both short range repulsive and longer-ranged attractive interactions, containing only two floating parameters, as opposed to at least four for a Lennard-Jones type potential. Yet, as demonstrated here, a square well-fluid can exhibit colligative properties and LLPS behavior in qualitative or semi-quantitative agreement with experimental measurement.

In addition, we point out that unlike simulations of Lennard-Jones fluids, square well-fluids at equilibrium may be simulated rapidly and precisely using the method of Discrete Molecular Dynamics (Proctor and Dokholyan, 2016) as utilized here. The algorithms employed in DMD are computationally far simpler and more rapid than those employed in conventional molecular or Brownian dynamics simulation, and avoid cumulative error resulting from the approximate numerical solution of differential equations.

Petsev et al. (2003) presented an alternative approach toward characterization of the composition dependence of intermolecular interaction in concentrated protein solutions. According to their treatment, the thermodynamic activity coefficient may be written as a sum of contributions from hard spherical repulsion and spherically symmetric “soft” interactions, just as in our Equation (12). The hard-sphere contribution is calculated according to the empirical Carnahan-Starling equation (Carnahan and Starling, 1969), and the soft contribution is written as a power series in the volume fraction

(19) $$\ln\gamma_{soft}=B_{2}^{*}\phi +B_{3}^{*}\phi^{2}+B_{4}^{*}\phi^{3}$$

This expression is comparable to our Equation (14), except that one additional higher-order term is included, and the coefficients are not defined with respect to any particular model of solute-solute interaction. At any given temperature, the values of the coefficients $B_{i}^{*}$ are determined by fitting the appropriate expressions for the concentration dependence of light scattering and osmotic pressure, calculated using the concentration dependent activity coefficient obtained as described above, to experimental measurement of concentration-dependent light scattering in the dilute one-phase regime, and measured values of $\phi_{dil}^{eq}$and $\phi_{conc}^{eq}$ in the two-phase regime. This approach works well, given the precise measurements of Petsev et al. (2003) of both concentration-dependent light scattering and the compositions of coexisting phases at multiple temperatures.

The treatment of Petsev et al may be characterized as a “top-down” approach, proceeding from experimental measurement to evaluation of the underlying intermolecular interaction potential. By contrast, our approach may be characterized as “bottom-up,” proceeding from a fully-defined model intermolecular interaction potential to a calculation of measurable concentration-dependent properties. The top-down approach, which requires a substantial quantity of high quality data obtained at multiple temperatures, can provide a detailed description of the temperature dependence of not only the potential of mean force, but also the enthalpic and entropic components of this potential for a specific protein under a particular set of experimental conditions (pH, buffer composition, ionic strength). In contrast, our objective is to explore the effect of systematically varying intermolecular interaction potential upon the concentration dependence of experimentally measurable solution properties. Unlike the top down analysis of Petsev et al. our treatment may be extended in a straightforward manner to solutions containing multiple macromolecular solutes. For example, the intermolecular interaction in a solution containing two macrosolutes may be characterized at a fixed temperature by Equation (10), with three values of *r* (i.e., *r*_11_, *r*_22_, and *r*_12_), three values of *L* and three values of ε^*^. We have already presented the results of a Kihara+ calculation of the composition dependence of ln γ_*i*_ of each of three solutes in a solution mixture (Hoppe and Minton, 2016), and the influence of hard and soft interactions upon selected chemical equilibria. We intend to extend this treatment to calculate the composition dependence of light scattering, osmotic pressure, and liquid-liquid phase equilibria in solutions of two macromolecular solutes.

In this work we have demonstrated how the Kihara+ model for effective interaction between solute molecules in a solution containing a single solute species can semi-quantitatively reproduce the concentration dependence of ln γ in a square well-fluid as calculated via rigorous computer simulation. The analytical model and the simulations yield similar predictions of the concentration dependence of light scattering and osmotic pressure. Finally, calculations of concentration-dependent light scattering, osmotic pressure, and liquid-liquid phase separation have been shown to closely resemble the corresponding properties measured experimentally in solutions of globular proteins. The Kihara+ model is particularly useful, as it is amenable to generalization to solutions containing more than one macrosolute species without recourse to increasingly more complex and compute-intensive simulations. Further development in this direction is underway. We thus conclude that the square well-potential, the simplest potential representing both steric repulsion and longer-ranged interactions, provides a conceptual basis for understanding the concentration-dependent equilibrium properties of globular proteins at high concentration, an initial and necessary step toward understanding the behavior of proteins in more complex cytomimetic media.

## Data Availability

The datasets generated for this study are available on request to the corresponding author.

## Author Contributions

All authors listed have made a substantial, direct and intellectual contribution to the work, and approved it for publication.

### Conflict of Interest Statement

The authors declare that the research was conducted in the absence of any commercial or financial relationships that could be construed as a potential conflict of interest.

## Footnotes

^1^Consider a solution containing a variety of solute molecules and solvent. If we fix the relative positions of just two macromolecular solute molecules of species *i* and *j* in this solution at a center-to-center distance r_ij_ and allow all of the other solute and solvent molecules to equilibrate at constant temperature and pressure, the solution reaches a minimum free energy subject to the positional constraints on the two specified molecules, which we will denote as *G*_*eq*_(*r*_*ij*_). We now define the potential of mean force to be

(1F1) $$U\left(r_{ij}\right)\equiv G_{eq}\left(r_{ij}\right)-G_{eq}(\infty )$$

Note that the value of *G*_*eq*_ at any center-to-center distance will depend upon the interactions between all of the solute and solvent molecules in the solution. Thus the potential of mean force is an effective potential of interaction between molecules *i* and *j within the solution* rather than the interaction between these molecules in a vacuum. McMillan and Mayer (McMillan and Mayer, 1945) demonstrated that the calculation of the osmotic pressure of a solution was formally identical to the calculation of the pressure of a gas, provided that the interaction between solute molecules was represented by the potential of mean force instead of the potential acting between molecules in the gas phase. The potential of mean force therefore takes into account solute-solute and solute-solvent interactions, but implicitly rather than explicitly.

^2^In our particular computations, we arbitrarily selected M = 70,000 g/mol and $\bar{v}$ = 0.73 cm^3^/g for the purpose of converting ϕ to *c*. The fundamental calculation of ln γ in a SW fluid depends upon the concentration of solute particles, the radius of the spherical particle, *r*, and the dimensionless quantities *L* and ε^*^. Since all distances may be scaled to units of *r*, volumes to units of *r*^3^, and concentrations to units of *r*^−3^, it is clear that ln γ is independent of values of mass and specific volume defined with respect to an arbitrarily selected set of units, since in principle they may also be scaled to molecular dimensions.

^3^Since ε^*^ ≡ ε/*kT*, it follows that a relative temperature may be defined: $T_{rel}=-kT/\varepsilon =-1/\varepsilon^{*}$.

^4^Increasing salt concentration generally damps out intermolecular electrostatic attraction and repulsion (Cohn and Edsall, 1943; von Hippel and Schleich, 1969).

^5^The rate of conversion of metastable to stable equilibrium may be so slow that the single-phase solution appears to be stable over a period of time exceeding the duration of practical experimental measurement. Measurement of the cloud point by reduction of temperature may indicate the spinodal rather than the binodal composition. The most certain way of determining the binodal or coexistence curve is to increase or decrease the concentration beyond the limits of metastability and then measure the concentrations of the protein in each of the two phases formed at equilibrium.
