# Supplementary material for: Non-specific Interactions Between Macromolecular Solutes in Concentrated Solution: Physico-Chemical Manifestations and Biochemical Consequences
Source: Front Mol Biosci. 2019 Mar 13;6:10. doi: 10.3389/fmolb.2019.00010 (PMC6424865; doi:10.3389/fmolb.2019.00010)
Supplement: Supplementary file 1 [file Data_Sheet_1.pdf]

Supplementary Information

Frontiers in Molecular Biosciences – doi: 10.3389/fmolb.2019.00010

Nonspecific interactions between macromolecular solutes in concentrated solution: physico-chemical manifestations and biochemical consequences

Travis Hoppe and Allen P. Minton*

Laboratory of Biochemistry and Genetics, National Institute of Diabetes and Digestive and Kidney Diseases, National Institutes of Health, Bethesda MD USA

* Correspondence: Allen P. Minton, minton@helix.nih.gov

Appendix 1: Fitting of 4th order polynomial to calculated dependence of $\ln\gamma$ on ϕ .

Appendix 2: Determination of phase boundaries via analysis of the concentration dependence of osmotic pressure

Appendix 1: Fitting of 4th order polynomial to calculated dependence of $\ln\gamma$ on ϕ .

Figure A1 - Symbols: results of square well simulation described in text, calculated for selected values of ε^* . Curves: best-fit of 4th order polynomial to correspondingly colored data sets. Panel A: *L* = 1.25. ε^* = 0 (black), -0.5 (blue), -1 (red), -1.5 (cyan). Panel B: *L* = 1.5. ε^* = 0 (black), -0.4 (blue), -0.8 (red), -1.2 (cyan). Best fit values of polynomial coefficients given in Table A1.

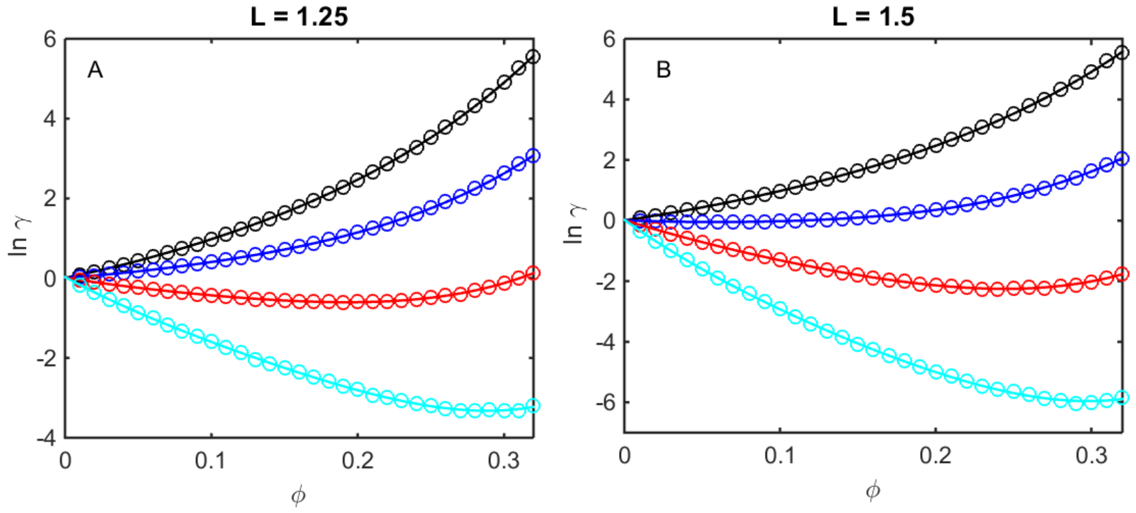

Table A1 - Best fit of polynomial to simulation results

y = P(1)*phi^4 + P(2)*phi^3 + P(3)*phi^2 + P(4)*phi + P(5)

eps*	P(1)	P(2)	P(3)	P(4)	P(5)
L = 1.25					
0	176.6072	-36.01012	24.60692	7.419891	0.007718136
-0.5	181.9077	-42.8264	17.24604	2.505072	0.007579137
-1	240.4802	-80.84007	20.97522	-5.961898	0.01360154
-1.5	443.068	-217.1545	54.90799	-20.02077	0.02678967
L = 1.5					
0	176.6072	-36.01012	24.60692	7.419891	0.007718136
-0.4	221.7903	-51.65119	20.16572	-2.027337	0.01005352
-0.8	324.7895	-118.1922	37.65649	-16.17029	0.0160313
-1.2	627.1891	-337.3784	101.4521	-36.9559	0.02663047

Appendix 2: Determination of phase boundaries via analysis of the concentration dependence of the osmotic pressure

For specified values of L and $\varepsilon *$, the dependence of osmotic pressure Π (in relative units) is calculated as a function of concentration as described in the text. When the calculated osmotic pressure exhibits non-monotonic behavior, the presence of a phase transition is indicated. The boundaries of the coexisting phases are calculated as follows. In the example shown in **Figure A2,** panel A, Π is plotted in units of λ (equation [4]) as a function of the unitless volume fraction ϕ. In **Figure A2,** panel B, Π is plotted as a function of the inverse concentration, which is proportional to the volume per molecule of solute.

Figure A2

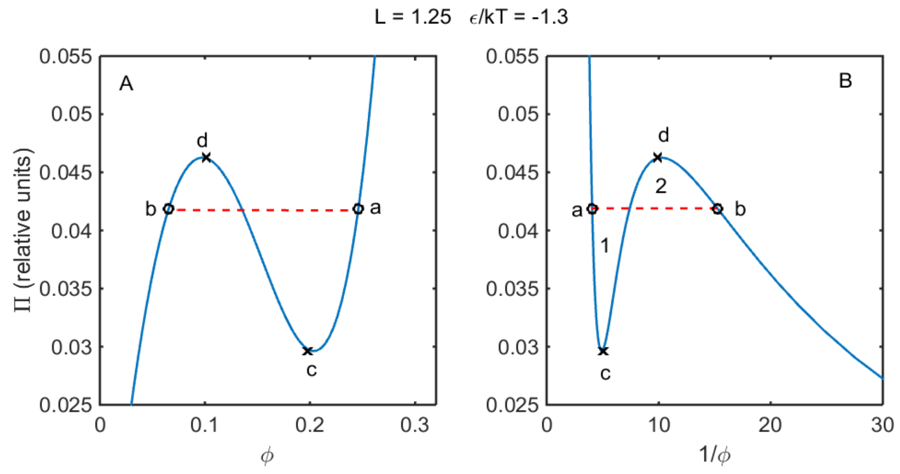

It follows from the solution theory of McMillan and Mayer (McMillan and Mayer 1945) that panel B is thermodynamically equivalent to a plot of pressure as a function of volume for a gas at constant temperature and fixed number of gas molecules, and may be similarly analyzed. According to the Maxwell equal-area construction (Eubank and Hall 1995), the compositions of the two coexisting phases at equilibrium are given by the points marked (a) and (b), which correspond to a value of the osmotic pressure such that the areas labeled 1 and 2 between the

calculated curve and the tie-line connecting (a) and (b) in Figure S1B are equal. For all compositions between those indicated between points (a) and (b), the osmotic pressure will remain constant at the height of the tieline connecting these points. The points labeled (c) and (d), represent the physical limits of a single phase solution. Compositions falling between the concentrations of points (b) and (d) on the low end, and points (a) and (c) on the high, end represent metastable states of a single phase solution that will ultimately demix to yield two phases with the equilibrium compositions indicated. Solutions with total concentrations between those of points (c) and (d) cannot exist as a single phase. Points (a) and (b) obtained at multiple values of ε/kT may be connected to yield a curve called the binodal or coexistence curve, and points (c) and (d) likewise obtained at multiple values of ε^* may be connected to yield a curve called the spinodal curve representing the limits of single-phase metastability.

References

Eubank, P. T. and Hall, K. R. (1995). Equal area rule and algorithm for determining phase compositions. *AIChE J.* 41, 924-927: doi: 10.1002/aic.690410419

McMillan, W. G. Jr. and Mayer, J. E. (1945). The statistical thermodynamics of multicomponent systems. *J. Chem. Phys.* 13, 276-305. doi: 10.1063/1.1724036
